# Quality and mechanical efficiency of automated knowledge‐based planning for volumetric‐modulated arc therapy in head and neck cancer

**DOI:** 10.1002/acm2.14588

**Published:** 2024-12-01

**Authors:** Sangutid Thongsawad, Sasikarn Chamchod, Kornkanok Chawengsaksopak, Wilai Masanga, Aphisara Deeharing, Sarinya Bawornpatarapakorn, Thitiwan Prachanukul, Chirapha Tannanonta, Nuntawat Udee

**Affiliations:** ^1^ Medical Physics Program Princess Srisavangavadhana College of Medicine Chulabhorn Royal Academy Bangkok Thailand; ^2^ Department of Radiation Oncology Chulabhorn Hospital Bangkok Thailand; ^3^ Department of Radiologic Technology Faculty of Allied Health Science Naresuan University Pitsanulok Thailand

**Keywords:** automated radiation treatment planning, head and neck cancers, knowledge‐based planning (KBP), RapidPlan, scripting application programming interface (API), volumetric‐modulated arc therapy (VMAT)

## Abstract

**Objectives:**

This study aimed to examine the effectiveness of the automated RapidPlan in assessing plan quality and to explore how beam complexity affects the mechanical performance of volumetric modulated arc therapy for head and neck cancers.

**Materials and methods:**

The plans were first generated using automated RapidPlan with scripting application programming interface (API) and then further refined through manual optimization (RP+MP) to improve plan quality. The quality of 20 plans was assessed, taking into account dose statistics and clinical plan acceptability. The impact of beam complexity on mechanical performance was analyzed using parameters such as leaf speed (LS), leaf acceleration (LA), mean‐field area (MFA), cross‐axis score (CAS), closed leaf score (CLS), small aperture score (SAS), and monitor units per control point (MU/CP). Patient‐specific quality assurance (PSQA) was conducted to determine differences between the RP+MP and original plans.

**Results:**

No differences in the heterogeneity index and conformity number were observed between the RP+MP and original plans. The RP+MP plan was superior to the original plan for sparing the left cochlea, left and right internal auditory canals, chiasm, and left optic nerve. Significant differences (*p* < 0.05) were identified in CAS, SAS_1_ _mm_, SAS_2_ _mm_, and SAS_10mm_. However, there was no significant difference in PSQA between the RP+MP and original plans. The RP+MP plan without any modifications was clinically acceptable in 45% of cases.

**Conclusion:**

The automated RP with scripting API followed by MP (RP+MP) yielded a high‐quality plan in terms of dose statistics and clinical acceptability. The RP+MP plan yielded a higher CAS and SAS compared with the original plan. Nevertheless, there was no significant difference in PSQA between the RP+MP and original plans.

## 1 INTRODUCTION

Volumetric‐modulated arc therapy (VMAT) is an advanced radiation treatment involving beam modulation during rotation of the gantry around the patient. VMAT delivers the beam by simultaneously adjusting MLC position, dose rate, and gantry speed. By offering increased degrees of freedom for beam modulation, VMAT enhances the conformal dose to the tumor while minimizing radiation exposure to normal organs.^1^ In cases of head and neck (HN) cancer, where the target and organs at risk (OAR) often have irregular shapes and overlapping areas, VMAT is particularly valuable. The technique facilitates the delivery of a high‐dose gradient and intricate beam modulation, addressing the complexity of such cases.^2^ VMAT proves to be an excellent choice for HN cancer treatment, contributing to enhanced tumor control and a reduction in radiation‐related complications.^1^, ^2^ Nevertheless, the optimization process is intricate and demands the expertise of skilled planners, which significantly impacts the quality and consistency of treatment plans. Recently, knowledge‐based planning (KBP) has gained widespread use in radiation therapy, offering a valuable tool to enhance both the quality and consistency of treatment plans.

Numerous studies have evaluated the efficacy of commercial KBP systems, such as RapidPlan (Varian Medical Systems, Palo Alto, California, USA), across diverse cancer types. The assessment of plan performance in the literature often hinges on two key metrics: statistical dose scores and clinically acceptable scores. Swamidas et al. and Castriconi et al.^2^, ^3^ investigated the application of RapidPlan in cervical cancer, revealing its superiority over manually generated plans in terms of tumor coverage and sparing OAR. In breast cancer, both the Frederick and Fogliata groups^3^, ^4^, ^5^ observed that RapidPlan consistently outperformed original plans in terms of consistency and quality.

For HN cancers, several studies^1^, ^6^, ^7^, ^8^ consistently reported favorable outcomes using RapidPlan, highlighting its ability to achieve both lower doses to normal organs and greater tumor coverage compared with original plans. Notably, certain authors^9^, ^10^, ^11^ underscored the clinical acceptability of plans generated by RapidPlan, subjecting them to evaluation by experienced radiation oncologists. The consensus from these evaluations suggests that RapidPlan can achieve clinical plan acceptability rates of 80%–90%.

The impact of plan complexity on beam delivery errors has been previously explored^12^, ^13^ using various complexity scores, such as beam variability, aperture variability, leaf speed (LS), leaf acceleration (LA), mean‐field area (MFA), mean asymmetry distance (MAD), cross‐axis score (CAS), closed leaf score (CLS), small aperture score (SAS), modulation complexity score, and monitor units (MU). For commercial KBP systems such RapidPlan, complexity has also been investigated,^14^ revealing that RapidPlan leads to reduced leaf travel and increased SAS compared to manually generated plans.

This study aimed to comprehensively examine the performance of automated RapidPlan followed by manual optimization (RP+MP) in terms of plan quality and assess the impact of beam complexity on mechanical performance in the context of VMAT for head and neck (HN) cancers. Plan quality was evaluated using dose statistics and clinical plan acceptability scores. The mechanical performance was assessed using parameters such as LS, LA, MFA, CAS, CLS, SAS, and MU per control point (MU/CP) for the original plan, RapidPlan only, and the RP+MP plan. Furthermore, a comparison was made between the patient‐specific quality assurance (PSQA) measures for the RP+MP and original plans.

## 2 METHOD

The optimization process used RapidPlan from version 16.1 of the Eclipse treatment planning system (Varian Medical Systems, Palo Alto, California, USA). The Eclipse scripting application programming interface (API) version 16.1 (Varian Medical Systems, Palo Alto, California, USA), based on the C# programming language, was used to develop the automated workflows for RapidPlan. Computed tomography (CT) images with a slice thickness of 3 mm were acquired using a Philips Bigbore scanner (Philips Medical Systems, Cleveland, Ohio, USA). All plans were delivered using a TrueBeam linear accelerator (Varian Medical Systems, Palo Alto, California, USA).

### 2.1 RapidPlan model training

One hundred HN VMAT plans from January 2010 to December 2015 were gathered from a single institution for RapidPlan model training. The dataset consisted of 34 nasopharyngeal, 33 oropharyngeal, and 33 hypopharyngeal and laryngeal cancer cases. All plans used the simultaneous integrated boost technique, with a prescription dose of 70 Gy (in 33 fractions) to the gross tumor, 60 Gy to high‐risk nodes, and 54 Gy to low‐risk nodes. In this study, the RapidPlan model included a posterior avoidance structure designed to restrict the dose level to 40–45 Gy around the spinal cord and brain stem. The RapidPlan model for HN used in this study is available as Supplementary material 1.

### 2.2 RapidPlan performance

We examined the performance of the automated RapidPlan followed by manual optimization, focusing on plan quality and the influence of complexity scores on the mechanical performance of beam delivery. We collected 20 historical plans outside the RapidPlan training dataset, consisting of eight nasopharyngeal, six oropharyngeal, and six hypopharyngeal and laryngeal cancer cases, to evaluate the performance of RP+MP. These original plans were manually optimized and delivered during the same period as the training dataset. Table 1 shows the characteristics of 20 historical plans.

**TABLE 1:** The characteristic of 20 historical plans.

		PTV volume (cm^3^)			
Patient#	Tumor site	Low‐risk nodes	Intermediate‐risk nodes	High‐risk nodes	Arc number
1	Nasopharynx	273.65	1027.88	424.34	4 full arcs
2	Nasopharynx	100.3	769.04	367.59	3 full arcs
3	Nasopharynx	256.81	855.8	303.32	4 full arcs
4	Nasopharynx	146.5	1137.1	589.02	4 full arcs
5	Oropharynx	220.15	724.92	471.51	4 full arcs
6	Oropharynx	90.13	314.17	354.01	4 full arcs
7	Oropharynx	91.33	755.11	258.38	3 full arcs
8	Hypopharynx	616.37	567.67	234.13	3 full arcs
9	Larynx	388.23	1060.86	602.87	4 full arcs
10	Hypopharynx	257.13	595.96	125.29	3 full arcs
11	Nasopharynx	259.02	649.13	187.86	4 full arcs
12	Nasopharynx	303.35	790.97	313.01	4 full arcs
13	Nasopharynx	84.46	672.80	247.33	3 full arcs
14	Oropharynx	999.51	61.92	123.2	4 full arcs
15	Nasopharynx	174.09	633.47	205.46	4 full arcs
16	Oropharynx	225.1	607.46	197.89	3 full arcs
17	Oropharynx	174.99	417.69	218.37	3 full arcs
18	Larynx	1008.32	176.71	60.85	3 full arcs
19	Hypopharynx	800.15	176.62	65.57	4 full arcs
20	Hypopharynx	664.42	116.62	35.99	3 full arcs

#### 2.2.1 The automated RapidPlan followed by manual optimization (RP+MP)

In our clinical practice, initial RapidPlan creation was followed by manual optimization (RP+MP) to enhance tumor coverage and minimize doses to OAR.

##### Initial automated RapidPlan with scripting API

**FIGURE 1:**
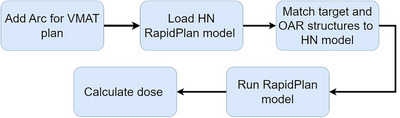
Framework of the application programming interface scripting used for automated RapidPlan generation.

The plan was initially created using scripting API, incorporating arc therapy beams into the plan. Beam parameters, such as machine type and energy, were specified. Subsequently, the RapidPlan model was applied, ensuring alignment of target and OAR structures between the plan and RapidPlan model. A volume dose calculation model (anisotropic analytical algorithm; AAA) was then assigned for calculating dose distribution. Finally, dose calculations were executed on the CT images. The goal of using scripting API initially is to streamline workflows and generate the optimal plan. Figure 1 depicts the framework of scripting API used for the automated generation of RapidPlan. The scripting API used to generate RapidPlan in this study is available as Supplementary material 2.

##### Manual optimization for plan improvement

After executing RapidPlan, medical physicists manually optimized the plans by following the dose constraint protocol (Table 2). Note that the photon optimizer (PO) algorithm operates across four multi‐resolution levels, progressively refining the arc resolution from coarse to fine detail. The manual optimization began with a continue the previous optimization step and was paused at level 3 to adjust the priority and objectives. This ensured compliance with specified constraints for organs at risk (OARs) and allowed adjustments for tumor dose coverage as needed. Furthermore, manual optimization was necessary to reduce the hot spot in OARs and ensure that the maximum hot spot in the body did not exceed 110%. Finally, the plan was normalized to ensure that at least 95% of the volume received 100% of the prescribed dose, if necessary.

**TABLE 2:** Dose constraints protocol of organs at risk (OAR) used for head and neck cancer treatment plans.

OAR	Dose constraint^a^
Brain stem	D_max_ < 54 Gy or 1% of target volume will receive dose < 60 Gy
Chiasm	D_max_ < 54 Gy or 1% of target volume will receive dose < 60 Gy
Cochlea	D_mean_ < 45 Gy
IAC	D_mean_ < 45 Gy
Eye	D_mean_ < 35 Gy
Len	D_max_ < 6–15 Gy
Optic nerve	D_max_ < 54 Gy or 1% of target volume will receive dose < 60 Gy
Parotid	D_mean_ < 26 Gy or one side will receive dose < 30 Gy
Spinal cord	D_max_ < 45 Gy

Abbreviation: IAC, internal auditory canal.

^a^ D_max_, maximum dose; D_mean_, mean dose.

#### 2.2.2 Plan quality

##### Dose statistics

To assess plan quality, dose statistics for the RP+MP and original plans were compared. Specifically, the dose difference between the plans was evaluated with respect to planning target volume (PTV) coverage and sparing of OAR. PTV coverage was determined using the conformity number (CN) and heterogeneity index (HI).^15^ The CN is defined by

(1) $$\mathrm{CN}=\frac{{\mathrm{PTV}}_{\mathrm{PD}}}{\mathrm{PTV}}\times \frac{{\mathrm{PTV}}_{\mathrm{PD}}}{\mathrm{PIV}},$$

where PTV_PD_ is the PTV volume receiving the prescription dose and PIV is the volume receiving the prescription dose. The HI is defined by

(2) $$\mathrm{HI}=\frac{D_{2}-D_{98}}{D_{50}},$$

where D_2_, D_50_, and D_98_ are the maximum doses that cover at least 2%, 50%, and 98% of the target volume, respectively. The OAR sparing was determined using dose constraints from the Radiation Therapy Oncology Group protocols 0225 and 0615,^16^, ^17^ as shown in Table 2. The Wilcoxon signed‐rank test with a 95% confidence level (*p* < 0.05) was used to identify significant differences between the RP+MP and original plans.

##### Clinical plan acceptability

In addition to the dose statistics, the dose distribution is evaluated slice‐by‐slice by radiation oncologists in clinical practice to approve a plan for treatment. Therefore, the clinical acceptability of the RP+MP plans in this study was blind‐reviewed by three radiation oncologists, each with more than 10 years of experience. They assigned scores for clinical plan acceptability following the criteria shown in Table 3.

**TABLE 3:** Clinical plan acceptability scores^9^ for evaluation of the automated RapidPlan followed by manual optimization (RP+MP).

Radiation oncologist scores	Score description
3	Acceptable as is
2	Prefer minor edits, but I would use this plan if necessary
1	Clinically acceptable plan, but I would require minor edits
0	Clinically unacceptable plan

#### 2.2.3 Impact of complexity score on mechanical performance

The plan complexity was assessed to investigate its effect on beam delivery accuracy. Beam complexity scores, including leaf speed (LS), leaf acceleration (LA), mean‐field area (MFA), cross‐axis score (CAS), closed leaf score (CLS), small aperture score (SAS), and monitor unit per control point (MU/CP), were calculated. Table 4 displays the description of beam complexity score used in this study.

**TABLE 4:** Description of beam complexity score used in this study.

No.	Beam complexity score	Description	Influence to beam complexity
1	Fraction of leaf speed in different ranges (LS _range_)	The proportion of leaf speed within the assigned range	A higher proportion of higher leaf speed increases beam complexity.
2	Fraction of Leaf acceleration in different ranges (LA _range_)	The proportion of leaf speed within the assigned range	A higher proportion of higher leaf acceleration increases beam complexity.
3	Mean‐field area (MFA)	Mean of the field area weighted according to the MU at each control point	A less MFA increases beam complexity.
4	Cross‐axis score (CAS)	The proportion of leaves crossing the center	A higher of leaves crossing increases beam complexity.
5	Closed leaf score (CLS)	The proportion of closed leaves	A higher of closed leaves increases beam complexity.
6	Small aperture score (SAS _aperture_)	The proportions of open leaf pairs separated by less than the assigned aperture	A higher proportion of small aperture increases beam complexity.
7	Monitor unit per control point (MU/CP)	The ratio of monitor unit to number of control points	A higher of MU/CP increases beam complexity.

In the case of LS and LA, the average proportions of LS and LA within various ranges were identified; however, only LS within the range of 16–20 mm/s and LA within the range of 160–200 mm/s^2^ were observed in the validated plans. Regarding the SAS, the proportions of open leaf pairs separated by distances less than 1 mm (SAS_1_ _mm_), 2 mm (SAS_2_ _mm_), 5 mm (SAS_5_ _mm_), and 10 mm (SAS_10_ _mm_) were evaluated.

We assessed the difference in beam complexity scores between the RP+MP and original plans. Additionally, we calculated the beam complexity scores for the RapidPlan alone (RP‐only) to determine whether RP+MP or RP‐only plans had a greater impact on beam complexity. The Wilcoxon signed‐rank test with a 95% confidence level (*p* < 0.05) was used to identify any significant differences in beam complexity scores between the RP+MP and RP‐only plans and between the RP+MP and original plans. To achieve this, scripting using MATLAB version 2019b (The Mathworks, Inc, Natick, Massachusetts, USA) was used to extract the beam complexity scores from DICOM plans. Additionally, the PSQA results for the RP+MP and original plans were compared.

At our institution, PSQA was conducted using images captured with the integrated aSi‐1000 electronic portal imaging device (EPID). The portal dose image prediction software from the Eclipse treatment planning system (version 13.6; Varian Medical Systems) was used to generate two‐dimensional image predictions. Subsequently, PSQA results were analyzed using gamma criteria of 3%/2 mm, 2%/2 mm, and 1%/1 mm (all at a 10% dose threshold).

## 3 RESULTS

### 3.1 Plan quality

Tables 5 and 6 show the statistical dose difference between RP+MP and original plans. For PTV (Table 5 and Supplementary material 3), no difference of both HI and CN values. In terms of OAR dose sparing (as indicated in Table 6 and Supplementary material 4), there were no significant differences observed for most organs, except for the optic chiasm, left optic nerve, right optic nerve, left lens, left cochlea, right cochlea, left internal auditory canal (IAC), and right IAC. The RP+MP plan demonstrated a significant reduction in dose (*p* < 0.05) for these specific organs.

**TABLE 5:** Tumor dose comparisons between RP+MP and original plans.

Target coverage		RP+MP^a^	Original plan^a^	*P value*
PTV low risk	CN	0.77 ± 0.04	0.75 ± 0.04	0.08
	HI	0.13 ± 0.04	0.14 ± 0.05	0.09
PTV intermediate risk	CN	0.83 ± 0.04	0.80 ± 0.06	0.06
	HI	0.19 ± 0.04	0.20 ± 0.03	0.58
PTV high risk	CN	0.83 ± 0.06	0.75 ± 0.15	0.06
	HI	0.08 ± 0.02	0.09 ± 0.03	0.13

Abbreviations: CN, conformity number; HI, heterogeneity index; PTV, planning treatment volume.

^a^ Data are mean ± standard deviation.

**TABLE 6:** OAR dose statistic comparisons between RP+MP and original plans.

OAR structure	Dose statistic^a^	RP+MP^b^	Original plan^b^	*P value*
Brain stem	D_1cc_	39.09 ± 6.32	39.90 ± 4.77	0.51
Optic chiasm	D_1%_	8.17 ± 7.33	12.78 ± 13.86	<0.05
Left optic nerve	D_1%_	13.46 ± 16.31	16.92 ± 18.98	<0.05
Right optic nerve	D_1%_	12.89 ± 16.34	15.94 ± 18.41	<0.05
Spinal cord	D_1cc_	38.13 ± 2.00	37.18 ± 2.93	0.17
Left lens	D_1%_	4.29 ± 2.89	4.54 ± 2.85	0.07
Right lens	D_1%_	4.49 ± 3.11	4.54 ± 2.85	<0.05
Left eye	D_mean_	5.05 ± 4.21	5.06 ± 3.88	0.39
Right eye	D_mean_	5.20 ± 4.41	5.30 ± 4.10	0.45
Left cochlea	D_mean_	21.33 ± 10.89	28.82 ± 12.52	<0.05
Right cochlea	D_mean_	22.24 ± 11.04	29.25 ± 12.67	<0.05
Left IAC	D_mean_	15.35 ± 10.28	24.57 ± 14.92	<0.05
Right IAC	D_mean_	15.78 ± 10.61	26.07 ± 14.96	<0.05
Left parotid gland	D_mean_	38.97 ± 6.09	37.30 ± 5.67	0.44
Right parotid gland	D_mean_	38.21 ± 7.36	36.75 ± 8.69	0.39

^a^ Data are mean ± standard deviation.

^b^ D_1cc_, dose (Gy) received by the OAR volume of 1 cm^3^; D_1%_, maximum dose (Gy) that covers at least 1% of the target volume.

**FIGURE 2:**
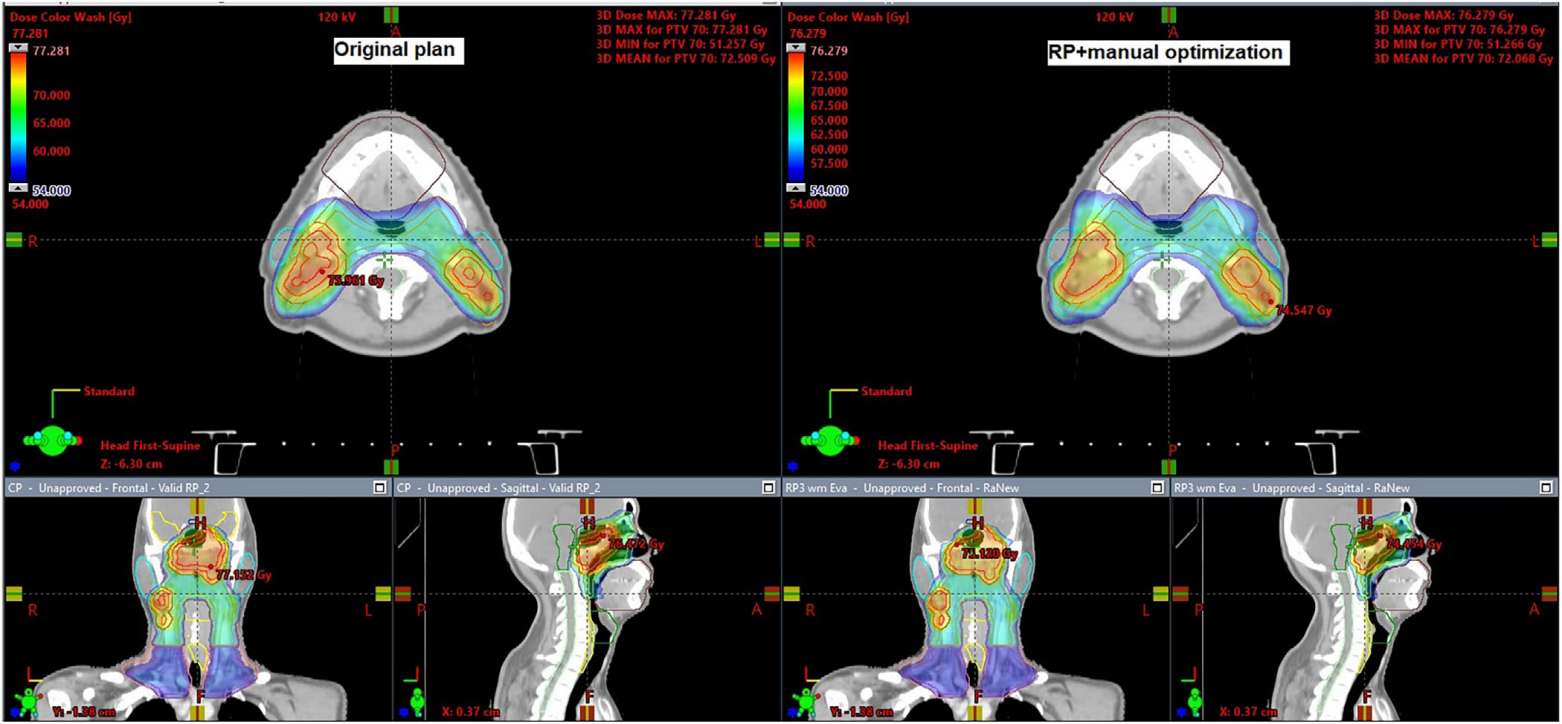
Example of a dose distribution comparison between the original and RP+MP plans.

Table 7 displays the clinical acceptability of the RP+MP plans. Forty‐five percent of the plans were deemed acceptable as is. Another 25% were considered to be acceptable as is, although minor edits would be preferred. Thirty percent of the plans were deemed to require minor edits before being acceptable. Notably, no plan was categorized as clinically unacceptable. Figure 2 shows an example of a dose distribution comparison between the original and RP+MP plans.

**TABLE 7:** Clinical acceptability scores for RP+MP plans.

Score description	Preferred plans/total plans
Acceptable as is	9/20 (45%)
Prefer minor edits, but I would use this plan if necessary	5/20 (25%)
Clinically acceptable plan, but I would require minor edits	6/20 (30%)
Clinically unacceptable plan	0/20 (0%)

### 3.2 Impact of complexity score on mechanical performance

Table 8 compares the complexity scores between the RP+MP and original plans. The RP+MP plan yielded significantly larger values of the CAS, SAS_1_ _mm_, SAS_2_ _mm_, and SAS_10_ _mm_ compared with the original plan (*p* < 0.05). Table 9 compares the plan complexity scores between RP+MP and RP‐only plans. For most complexity metrics, the RP+MP plans produced significantly lower scores compared to the RP‐only plans. However, the MFA score was higher in the RP+MP plans than in the RP‐only plans. This demonstrates that RP alone produces plans with greater complexity than RP+MP. Using the gamma passing rate as a PSQA measure, no significant difference between the RP+MP and original plans was found (Table 10).

**TABLE 8:** Complexity score comparisons between RP+MP and original plans.

	RP+MP^a^	Original plan^a^	
Plan complexity score	Mean ± SD (*Min‐Max*)	Mean ± SD (*Min‐Max*)	*P value*
LS_A16–20_	0.16 ± 0.21 (*0.12*–*0.29*)	0.2 ± 0.03 (*0.12*–*0.25*)	0.6262
LS_B16–20_	0.21 ± 0.03 (*0.16*–*0.31*)	0.21 ± 0.03 (*0.16*–*0.27*)	0.3919
LA_A160–200_	0.23 ± 0.04 (*0.17*–*0.31*)	0.22 ± 0.03 (*0.15*–*0.28*)	0.1356
LA_B160–200_	0.23 ± 0.04 (*0.17*–*0.3*)	0.23 ± 0.03 (*0.17*–*0.27*)	0.1767
MFA (mm^2^)	6298.32 ± 1606.29 (*4393.96*–*13 162.1*)	6631.42 ± 1726.17 (*3495.53*–*10 453.74*)	0.2204
CAS	0.85 ± 0.53 (*0.24‐2.29*)	0.73 ± 0.45 (*0.28‐2.29*)	(<0.05) 0.029
CLS	0.07 ± 0.04 (*0.01*–*0.2*)	0.07 ± 0.05 (*0*–*0.2*)	0.1717
SAS_1mm_	64.1 ± 12.52 (*44.18*–*93.93*)	62.37 ± 14.34 (*40.12*–*96.87*)	(<0.001) 0.0003
SAS_2mm_	65.61 ± 12.26 (*46.47*–*95.3*)	64.29 ± 14.52 (*41.98*–*99.53*)	(<0.05) 0.0112
SAS_5mm_	73.16 ± 11.03 (*58.87*–*101.53*)	70.93 ± 15.56 (*49.25*–*105.35*)	0.099
SAS_10mm_	87.06 ± 9.77 (*66.6*–*108.68*)	82.51 ± 16.12 (*61.65*–*111.75*)	(<0.05) 0.046
MU/CP	1.02 ± 0.19 (*0.66*–*1.37*)	1.01 ± 0.21 (*0.73*–*1.42*)	0.6945

Abbreviations: CAS, cross‐axis score; CLS, closed leaf score; LA_X160–200_, average proportion of leaf accelerations within the 160–200 mm/s^2^ for leaf bank X; LS_X16–20_, average proportion of leaf speeds within the 16–20 mm/s range for leaf bank X; MAD, mean asymmetry distance; MFA, mean‐field area; MU/CP, monitor units per control point; SAS*_x_*_mm_, proportion of open leaf pairs separated by less than *x* mm.

^a^ Max, maximum; Min, minimum; SD, standard deviation.

**TABLE 9:** Complexity score comparisons between the RP+MP plan and RapidPlan alone (RP‐only).

	RP+MP	RP‐only	
Plan complexity score	Mean ± SD (*Min‐Max*)	Mean ± SD (*Min‐Max*)	*P value*
LS_A16–20_	0.16 ± 0.21 (*0.12*–*0.29*)	0.23 ± 0.05 (*0.15*–*0.32*)	<0.001
LS_B16–20_	0.21 ± 0.03 (*0.16*–*0.31*)	0.24 ± 0.04 (*0.18*–*0.31*)	<0.001
LA_A160–200_	0.23 ± 0.04 (*0.17*–*0.31*)	0.25 ± 0.05 (*0.17*–*0.33*)	<0.001
LA_B160–200_	0.23 ± 0.04 (*0.17*–*0.3*)	0.25 ± 0.05 (*0.18*–*0.32*)	<0.001
MFA (mm^2^)	6,298.32 ± 1,606.29 (*4393.96*–*13 162.1*)	5251.54 ± 1581.99 (*3090.5*–*10 636.87*)	<0.001
CAS	0.85 ± 0.53 (*0.24*–*2.29*)	1.05 ± 0.63 (*0.35*–*2.4*)	(<0.05) 0.0024
CLS	0.07 ± 0.04 (*0.01*–*0.20*)	0.09 ± 0.04 (*0.01*–*0.21*)	<0.001
SAS_1mm_	64.1 ± 12.52 (*44.18*–*93.93*)	67.53 ± 11.26 (*48.05*–*97.1*)	<0.001
SAS_2mm_	65.61 ± 12.26 (*46.47*–*95.3*)	70.18 ± 10.88 (*51.37*–*101.18*)	<0.001
SAS_5mm_	73.16 ± 11.03 (*58.87*–*101.53*)	81.21 ± 9.43 (*67.42*–*109.58*)	<0.001
SAS_10mm_	87.06 ± 9.77 (*66.6*–*108.68*)	97.99 ± 9.71 (*78.05*–*118.15*)	<0.001
MU/CP	1.02 ± 0.19 (*0.69*–*1.37*)	1.22 ± 0.31 (*0.73*–*2.11*)	<0.001

**TABLE 10:** Comparison of gamma passing rates between the RP+MP and original plans using different gamma criteria.

	Gamma passing rate								
	RP+MP				Original plan				
Gamma criteria	Max	Min	Mean	SD	Max	Min	Mean	SD	*P value*
1%/1 mm	92.8	67.1	82.87	5.038	91.7	66.3	81.21	5.595	0.109
2%/2 mm	99.6	97.3	98.83	0.555	99.8	96.8	98.90	0.655	0.465
3%/2 mm	99.9	96.3	98.59	1.057	100	95.4	98.73	1.227	0.421

## 4 DISCUSSION

The conventional manual workflow for RapidPlan generation involves the following steps and interactions:

1. Inserting the plan and adding initial arc beams
2. Loading the RapidPlan model and matching structures to the model
3. Running the RapidPlan model
4. Calculating the dose distribution on CT images

We implemented a scripting API approach to automate these steps (Figure 1). This significantly reduces the planner's interaction with the treatment planning system, thereby minimizing the time required for RapidPlan generation and reducing the potential for errors during planning. In our clinic, manual plan optimization for HN cases typically takes approximately 2–7 h, depending on the plan's complexity and the planner's experience. The RP+MP process demonstrates a semi‐automated planning approach that significantly reduces planning time. The automated RapidPlan step takes about 5 min, including tasks such as inserting the plan, setting beam arcs, configuring RapidPlan parameters, matching structure names between the plan and the RapidPlan model, running the RapidPlan optimization, and performing dose calculations on CT images. Following this, manual optimization to improve plan quality takes an additional 20–40 min, depending on plan complexity. Therefore, the entire RP+MP process takes approximately 25–45 min. However, manual optimization is still required in some cases to enhance plan quality, such as reducing hotspots in OARs and minimizing cold spots in target areas.

In this study, we designed a RapidPlan model specifically for HN cancers. Our methodology involved compiling a diverse dataset covering multiple cancer sites within the HN region. The primary focus was on crafting a model capable of accommodating all types of HN treatment plans. As a result, the size of training dataset used in this study was larger than that used in other researches.^18^, ^19^

Liu et al.^20^ demonstrated that a two‐step optimization method enhances plan quality and diminishes inter‐planner variability for HN cases, yielding clinical plans with 57.5% acceptability. In comparison, our study revealed that 45% of RP+MP plans were acceptable without any edits. Plans were mostly scored 1 or 2 by the radiation oncologists because of low tumor dose coverage and poor dose conformity, whereas none of these plans had issues regarding OAR dose sparing. Therefore, one manual optimization was generally required to improve target coverage and dose conformity in our clinic. For the plan evaluation workflow in our department, the dose statistics were first determined by using clinical goals, and then, the dose distribution was determined and overlaid on CT images slice‐by‐slice. Hot spots at the boundary between the target and OAR were minimized to avoid radiation complications from plan uncertainty. The maximum dose in a hot spot should be less than 110% of the prescribed dose. The stringent criteria and thorough evaluation make it challenging for a plan to be accepted for treatment without any edits.

Note that beam complexity is not typically used clinically in our department. In this study, beam complexity was calculated to determine its influence on the mechanical performance of the machine. We also calculated beam complexity for monitoring purposes and future research. Specifically, we selected cases with high beam complexity for PSQA measurements and used beam complexity to predict PSQA results.

To our knowledge, this research represents the first investigation of the impact on beam complexity of RP‐only and RP+MP plans. The RP‐only plans uniquely impacted beam complexity by generating a larger LS, LA, CAS, CLS, SAS, and MU/CP compared with the RP+MP plan. Differences between RP+MP and RP alone were observed, revealing that the stricter dose constraints and priorities were evident in RP alone. This may lead to higher beam complexity in RP alone compared to RP+MP.

The beam complexity was compared between the RP+MP and the original plans. The results showed that CAS, SAS_1_ _mm_, SAS_2_ _mm_, and SAS_10_ _mm_ scores were higher in the RP+MP plans compared to the original plans. This finding is similar to that in previous studies,^10^, ^14^, ^21^ which observed that RapidPlan introduces beam complexity by requiring greater usage of small leaf widths and greater MU compared with the manual plan. In this study, although the beam complexity of the initial RapidPlan was lower after manual optimization by a medical physicist, certain complexity scores, namely the CAS, SAS_1_ _mm_, SAS_2_ _mm_, and SAS_10_ _mm_, remained higher than those in the original plan. However, the PSQA revealed no significant difference between the RP+MP and original plans. This finding aligned closely with that of Tamura et al.,^10^ who measured PSQA with film dosimetry and ArcCheck, finding no significant difference between the RapidPlan and the original plan when using a gamma criterion of greater than 2%/2 mm. Furthermore, our study extended the evaluation to include a more stringent gamma criterion of 1%/1 mm to thoroughly explore potential errors. Despite this increased scrutiny, the results revealed no significant difference between the RP+MP and original plans.

In this study, PSQA utilized EPID measurements, introducing concerns about the potential inaccuracies associated with EPID measurements. To mitigate the impact of accumulated dose effects on EPIDs, a dose calibration was performed before each measurement. Furthermore, the influence of backscatter from the arm support was corrected using Varian's preconfigured portal dosimetry package along with a two‐dimensional profile correction image.^22^

## 5 CONCLUSION

The automated RP with scripting API followed by MP (RP+MP) proved effective in generating high‐quality plans exhibiting comparable tumor coverage and greater sparing of OAR compared with the original plan. Among the 20 plans examined, the RP+MP plan without any modifications was clinically acceptable in 45% of cases, and none of the plans was categorized as clinically unacceptable. Notably, the RP+MP plan demonstrated a higher CAS and SAS compared with the original plan. However, there was no significant difference in PSQA results between RP+MP and the original plan.

## AUTHOR CONTRIBUTIONS

Sangutid Thongsawad involved in conceptualization, methodology, investigation, writing original draft, supervision, project administration and visualization. Sasikarn Chamchod, Sarinya Bawornpatarapakorn, and Thitiwan Prachanukul involved in methodology, clinical plan evaluation of RapidPlan performance, and editing the original draft. Kornkanok Chawengsaksopak involved in data curation, formal analysis, investigation, and writing original draft. Wilai Masanga and Aphisara Deeharing involved in conceptualization, methodology, investigation, supervision, and editing the original draft. Chirapha Tannanonta and Nuntawat Udee involved in conceptualization, editing the original draft, supervision, project administration.

## CONFLICT OF INTEREST STATEMENT

The authors declare no conflicts of interest.

## Supporting information



SUPPORTING INFORMATION

SUPPORTING INFORMATION

SUPPORTING INFORMATION

SUPPORTING INFORMATION

## Data Availability

The data that support the findings of this study are available from the corresponding author upon reasonable requests.
