# Supplementary material for: Quality and mechanical efficiency of automated knowledge‐based planning for volumetric‐modulated arc therapy in head and neck cancer
Source: J Appl Clin Med Phys. 2024 Dec 1;26(2):e14588. doi: 10.1002/acm2.14588 (PMC11799909; doi:10.1002/acm2.14588)
Supplement: Supplementary file 1 — SUPPORTING INFORMATION [file ACM2-26-e14588-s003.docx]

**Supplementary material 1.** Link to download RapidPlan model for head and neck cancers; [RapidPlan model](https://chulabhornroyalacademy-my.sharepoint.com/:f:/g/personal/sangutid_tho_cra_ac_th/Eqbz23LcRidNisLxfXTrRmQBUJDeILlte6d0ddEfadA3ng?e=Gl12kP)
