# Supplementary material for: Quality and mechanical efficiency of automated knowledge‐based planning for volumetric‐modulated arc therapy in head and neck cancer
Source: J Appl Clin Med Phys. 2024 Dec 1;26(2):e14588. doi: 10.1002/acm2.14588 (PMC11799909; doi:10.1002/acm2.14588)
Supplement: Supplementary file 2 — SUPPORTING INFORMATION [file ACM2-26-e14588-s004.docx]

**Supplementary material 2.** Scripting API for generating RapidPlan.

//---------------------------------------------------------------------------------------------

/// THE SOFTWARE IS PROVIDED "AS IS", WITHOUT WARRANTY OF ANY KIND, EXPRESS OR IMPLIED, INCLUDING

/// BUT NOT LIMITED TO THE WARRANTIES OF MERCHANTABILITY, FITNESS FOR A PARTICULAR PURPOSE AND

/// NONINFRINGEMENT. IN NO EVENT SHALL THE AUTHORS OR COPYRIGHT HOLDERS BE LIABLE FOR ANY CLAIM,

/// DAMAGES OR OTHER LIABILITY, WHETHER IN AN ACTION OF CONTRACT, TORT OR OTHERWISE, ARISING FROM,

/// OUT OF OR IN CONNECTION WITH THE SOFTWARE OR THE USE OR OTHER DEALINGS IN THE SOFTWARE.

/// </copyright>

/// <Versions>

/// This script was developed using Varian ESAPI v15.6 and Varian Eclipse v15.6

/// </Versions>

//---------------------------------------------------------------------------------------------

using System;

using System.Linq;

using System.Text;

using System.Windows;

using System.Collections.Generic;

using System.Reflection;

using System.Runtime.CompilerServices;

using VMS.TPS.Common.Model.API;

using VMS.TPS.Common.Model.Types;

// TODO: Replace the following version attributes by creating AssemblyInfo.cs. You can do this in the properties of the Visual Studio project.

[assembly: AssemblyVersion("1.0.0.1")]

[assembly: AssemblyFileVersion("1.0.0.1")]

[assembly: AssemblyInformationalVersion("1.0")]

// TODO: Uncomment the following line if the script requires write access.

[assembly: ESAPIScript(IsWriteable = true)]

namespace VMS.TPS

{

public class Script

{

public Script()

{

}

[MethodImpl(MethodImplOptions.NoInlining)]

public void Execute(ScriptContext context /*, System.Windows.Window window, ScriptEnvironment environment*/)

{

// Disclaimer: The code presented is intended for educational purposes only and it is probably not be the most efficient or elegant.

// There are multiple ways to perform the same logic or actions

// Code execution checks are not included for clarity

#region Create course/plan/fields

// Initial variables

// Lists for Structure ID matching

List<string> oars_id = new List<string>() { }; // Initialize using structure template structures IDs

List<string> model_oars_id = new List<string>() { }; // Initilize using estimation model structures IDs

// Dictionaries for model loading

Dictionary<string, string> matches = new Dictionary<string, string>();

Dictionary<string, DoseValue> levels = new Dictionary<string, DoseValue>();

// Check for open patient and structure set in context

if (context.Patient == null || context.StructureSet == null)

{

MessageBox.Show("Please load a patient, and structure set before running this script.", msg_caption, MessageBoxButton.OK, MessageBoxImage.Exclamation);

return;

}

// Open structure set

StructureSet c_ss = context.StructureSet;

// Start modifications

context.Patient.BeginModifications();

// Enter a new course, plans and fields

Course course = context.Patient.AddCourse();

course.Id = "RapidPlan";

// Add external beam plana and assign ID

ExternalPlanSetup eps = course.AddExternalPlanSetup(c_ss);

eps.Id = "RP";

// Parameters for beams

VVector isocenter = new VVector(c_ss.Image.UserOrigin.x, c_ss.Image.UserOrigin.y, c_ss.Image.UserOrigin.z);

var b_params = new ExternalBeamMachineParameters("MachineID", "6X", 600, "ARC", null); // Fill the Linac Machine ID according to your Machine

// Add arc beams

Beam arc_field1 = eps.AddArcBeam(b_params, new VRect<double>(-80, -120, 80, 120), 350, 179, 181, GantryDirection.CounterClockwise, 0, isocenter);

Beam arc_field2 = eps.AddArcBeam(b_params, new VRect<double>(-80, -120, 80, 120), 10, 181, 179, GantryDirection.Clockwise, 0, isocenter);

Beam arc_field3 = eps.AddArcBeam(b_params, new VRect<double>(-60, -120, 60, 120), 355, 179, 181, GantryDirection.CounterClockwise, 0, isocenter);

Beam field4 = eps.AddSetupBeam(b_params, new VRect<double>(-100, -100, 100, 100), 0, 270, 0, isocenter);

Beam field5 = eps.AddSetupBeam(b_params, new VRect<double>(-100, -100, 100, 100), 0, 180, 0, isocenter);

Beam field6 = eps.AddSetupBeam(b_params, new VRect<double>(-100, -100, 100, 100), 0, 0, 0, isocenter);

arc_field1.Id = "A1";

arc_field2.Id = "A2";

arc_field3.Id = "A3";

field4.Id = "Setup S270 kV";

field5.Id = "Setup S180 kV";

field6.Id = "Setup S0 CBCT";

// Create and add DRRs

var drr = new DRRCalculationParameters(500);

drr.SetLayerParameters(0, 1, -100, 1000, -1000, 1000);

arc_field1.CreateOrReplaceDRR(drr);

arc_field2.CreateOrReplaceDRR(drr);

arc_field3.CreateOrReplaceDRR(drr);

field4.CreateOrReplaceDRR(drr);

field5.CreateOrReplaceDRR(drr);

field6.CreateOrReplaceDRR(drr);

#endregion

#region Load RapidPlan(TM) model

// Add targets to IDs dictionary. Target IDs can be obtained programatically

// This the example of target ID from structure that matche to your RP model strucure

matches.Add("PTV 70", "PTV_High");

matches.Add("PTV 59.4", "PTV_Intermediate");

matches.Add("PTV 54", "PTV_Low");

//matches.Add("opt_ptv_2", "PTV_2");

//matches.Add("opt_ptv_3", "PTV_3");

matches.Add("Brain stem", "BrainStem");

matches.Add("z BS+0.5", "PRV_BrainStem");

matches.Add("z Cord+0.5", "PRV_SpinalCord");

matches.Add("Optic chiasm", "Optic Chiasm");

matches.Add("Optic n._Rt", "OpticNerve_R");

matches.Add("Optic n._Lt", "OpticNerve_L");

matches.Add("Parotid_Lt", "Parotid_L");

matches.Add("Parotid_Rt", "Parotid_R");

matches.Add("Cochlea_Lt", "Cochlea_L");

matches.Add("Cochlea_Rt", "Cochlea_R");

matches.Add("Eye_Lt", "Eye_L");

matches.Add("Eye_Rt", "Eye_R");

matches.Add("Len_Lt", "Len_L");

matches.Add("Len_Rt", "Len_R");

matches.Add("Lt IAC", "IAC_L");

matches.Add("Rt IAC", "IAC_R");

matches.Add("Mandible", "Mandible");

matches.Add("Temporal Rt", "TemporalLobe_R");

matches.Add("Temporal Lt", "TemporalLobe_L");

matches.Add("Larynx", "Larynx");

matches.Add("Esophagus", "Esophagus");

matches.Add("Pharynx", "Pharynx");

matches.Add("Mucosa", "Mucosa");

matches.Add("TM-joint_Lt", "TMJoint_L");

matches.Add("TM-joint_Rt", "TMJoint_R");

matches.Add("zAV40", "zAV40");

matches.Add("zAV45", "zAV45");

matches.Add("zorg80", "zRing_80");

matches.Add("zorg90", "zRing_90");

// Add targets to dose level dictionary. Dose values are an example

levels.Add("PTV 70", new DoseValue(70, "Gy"));

levels.Add("PTV 59.4", new DoseValue(59.4, "Gy"));

levels.Add("PTV 54", new DoseValue(54, "Gy"));

//levels.Add("opt_ptv_2", new DoseValue(190, "cGy"));

//levels.Add("opt_ptv_3", new DoseValue(180, "cGy"));

// Add OAR IDs to dictionary. Lists in first section need to be initialized prior running this section

for (int i = 0; i < oars_id.Count(); i++)

{

Structure model_add = c_ss.Structures.FirstOrDefault(x => x.Id == oars_id.ElementAt(i));

if (model_add != null)

{

matches.Add(oars_id.ElementAt(i), model_oars_id.ElementAt(i));

}

}

// Set the prescripton dose in Eclipse. Entered dose is an example

eps.SetPrescription(33, new DoseValue(2.12, "Gy"), 1);

// Set DVH estimation algorithm to be employed

eps.SetCalculationModel(CalculationType.DVHEstimation, "DVH Estimation Algorithm [17.0.1]"); // Fill the DVH Estimation Algorithm according to your version

// Calculate DVH Estimates using a RapidPlan model

eps.CalculateDVHEstimates("RP Model ID", targetDoseLevels: levels, structureMatches: matches); // Fill the RP model ID according to your RP model

// Add NTO

eps.OptimizationSetup.AddNormalTissueObjective(100, 3, 95, 50, 0.2);

#endregion

#region Optimize and Calculate plan

// Set optimization and calculation models

eps.SetCalculationModel(CalculationType.PhotonVolumeDose, "AAA_17010"); // Fill the calculation algorithm ID according to your algorithm

// Calculate DVH Estimates using a RapidPlan model

eps.SetCalculationModel(CalculationType.PhotonVMATOptimization, "PO_17010");//Fill the optimization algorithm ID according to your algorithm

// Example of optimization option set by code

eps.SetCalculationOption("PO_17010", "AirCavityCorrection", "On");//Fill the optimization option for AirCavityCorrection

// Run optimization

eps.OptimizeVMAT();

#endregion

// End of execution

MessageBox.Show("Script has finalized execution correctly.", msg_caption, MessageBoxButton.OK, MessageBoxImage.Exclamation);

}

}

}
