# Supplementary material for: Quality and mechanical efficiency of automated knowledge‐based planning for volumetric‐modulated arc therapy in head and neck cancer
Source: J Appl Clin Med Phys. 2024 Dec 1;26(2):e14588. doi: 10.1002/acm2.14588 (PMC11799909; doi:10.1002/acm2.14588)
Supplement: Supplementary file 3 — SUPPORTING INFORMATION [file ACM2-26-e14588-s001.docx]

**Supplementary materials 3.** Box plot illustrating the dose difference between RP+MP and original plans for **a**) Conformity Number (CN) and **b**) Homogeneity Index (HI).


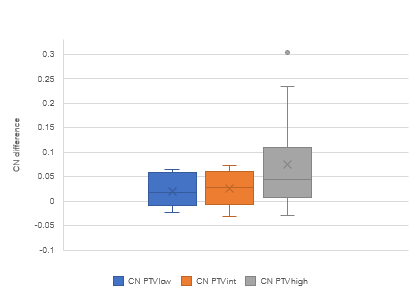

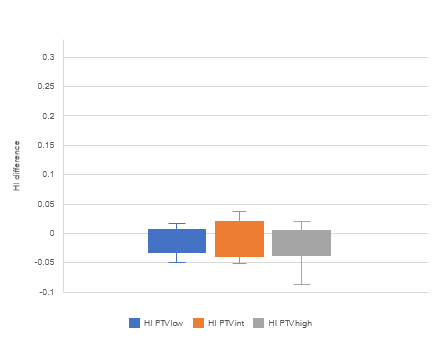


b)

a)
